# A Pediatric Primary Cardiac Spindle Cell Neoplasm With a Rare PDGFRA::USP8 Gene Fusion: A Case Report

**DOI:** 10.1177/10935266231221903

**Published:** 2024-02-24

**Authors:** Ariel Gershon, Anita Nagy, Gino R. Somers, Shi-Joon Yoo, Furqan Shaikh, Osami Honjo, Robert Siddaway, Haiying Chen

**Affiliations:** 1 Medical Genetics and Genomics, University of Toronto, Toronto, ON, Canada; 2 Division of Pathology, The Hospital for Sick Children, Toronto, ON, Canada; 3 Department of Laboratory Medicine and Pathobiology, University of Toronto, ON, Canada; 4 Department of Diagnostic Imaging, The Hospital for Sick Children, Toronto, ON, Canada; 5 Department of Medical imaging, University of Toronto, ON, Canada; 6 Department of Paediatrics, University of Toronto, ON, Canada; 7 Division of Haematology/Oncology, The Hospital for Sick Children, Toronto, ON, Canada; 8 Division of Cardiovascular Surgery, The Hospital for Sick Children, Toronto, ON, Canada; 9 Department of Surgery, University of Toronto, ON, Canada

**Keywords:** pediatric, cardiac, PDGFRA, USP8, sarcoma, gene fusion

## Abstract

We report a case of a primary cardiac spindle cell neoplasm with concerning histological features and a rare *PDGFRA::USP8* gene fusion in a 3 year old boy. The patient presented with a large cardiac mass predominantly in the right ventricle, originating from the ventricular septum. The mass was resected with grossly negative margins. Pathology revealed an unclassified spindle cell neoplasm with a *PDGFRA::USP8* gene fusion. This gene fusion has only been previously reported twice in the medical literature, one in a pediatric cardiac sarcoma and the other in an abdominal soft tissue tumor in an adult woman. The patient is alive and well with no evidence of recurrence 11 months after excision.

## Introduction

Primary cardiac tumors are rare in the pediatric population, with an estimated incidence of 0.0017–0.28.^1^ The majority of primary cardiac tumors in children are benign, with rhabdomyoma the most common. Approximately 10% of primary cardiac tumors are malignant.^1^ The rarity of pediatric primary cardiac tumors makes the diagnosis and treatment of these tumors challenging.

We report a case of a pediatric cardiac spindle cell neoplasm with an uncertain histogenesis and concerning histological features associated with a rare *PDGFRA*::*USP8* gene fusion. This case highlights the importance of thorough molecular investigation and the value of a multidisciplinary approach in managing such rare tumors.

## Case Report

**Figure 1.**
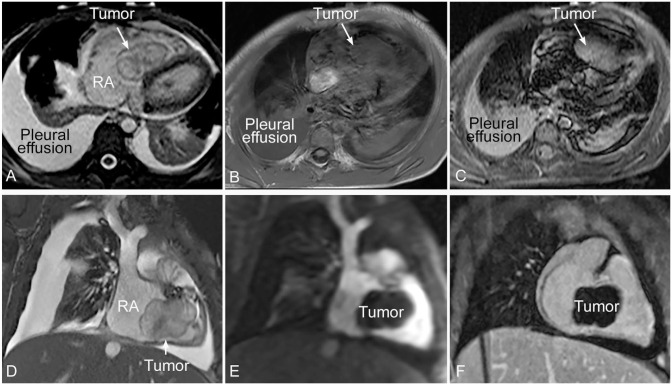
MRI images showing encapsulated mobile tumor in the cavity of the right atrium and ventricle across the tricuspid valve. It is attached to the inlet part of the ventricular septum. The tumor shows heterogeneous signal intensity on steady state free precession (SSFP) images (A and D). It is a low signal intensity lesion at T1-wieghted imaging (B) and high signal intensity lesion at T2-weighted imaging (C). The lesion does not show significant enhancement at perfusion imaging (E) and late gadolinium enhancement imaging (F).

A previously healthy 3 year old boy presented to the emergency department with mild abdominal pain and a low grade fever. He was found to have abdominal distention, ascites, and mild limb edema. Echocardiography revealed a large intracardiac right ventricular tumor, causing obstruction of the tricuspid valve inflow with secondarily dilated right atrium, inferior vena cava, and hepatic vein. Magnetic Resonance Imaging (MRI) was performed (Figure 1), demonstrating a well-defined lobulated mass (4.5 cm) inside right atrium and right ventricle. The mass was mobile across the tricuspid valve with its small part appearing attached to the inlet part of the ventricular septum. It showed low signal intensity at T1-weighted imaging and high signal intensity at T2-weighted imaging. Both T1 and T2 relaxation times of the tumor were prolonged as compared to the normal septal myocardium. The tumor did not show significant enhancement at perfusion imaging and late gadolinium enhancement imaging.

Surgical tumor resection was undertaken. The tumor was found to originate from the ventricular septum and was firmly adherent to the septal leaflet, distorting the tricuspid valve anatomy. The majority of the tumor was resected, with the septal leaflet of the tricuspid valve margin intentionally left positive to preserve the conduction system. The tricuspid valve was repaired with an autologous pericardial patch.

**Figure 2.**
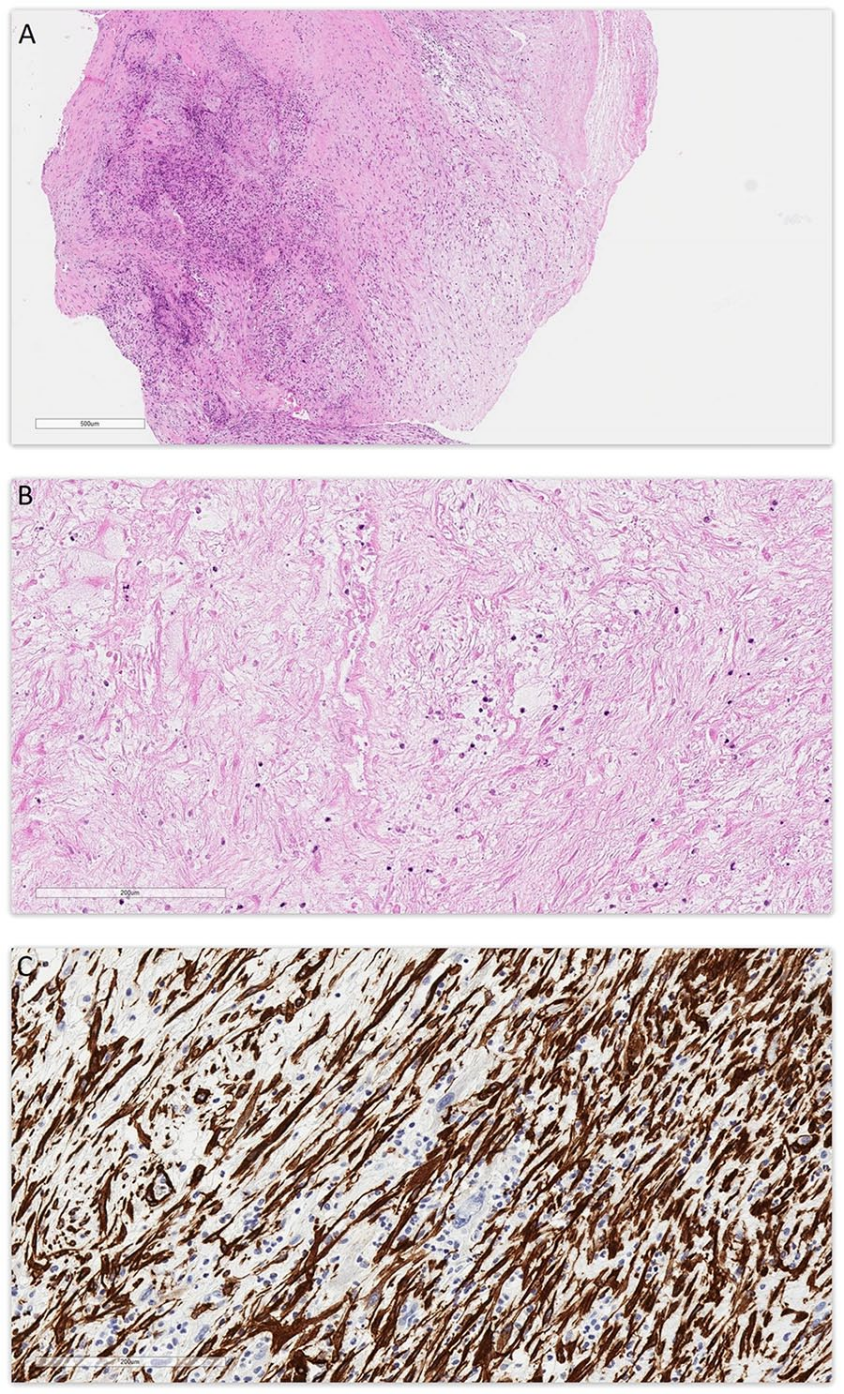
(A) Low magnification view showing tumor with variable low to moderate cellularity and fibromyxoid background; Stain: hematoxylin and eosin; Original magnification: 40×; Scale bar: 600 µm. (B) Higher magnification view showing coagulative type necrosis in tumor; Stain: hematoxylin and eosin; Original magnification: 200×; Scale bar: 200 µm. (C) Immunohistochemistry for smooth muscle actin (SMA); Original magnification: 200×; Scale bar: 200 µm.

**Figure 3.**
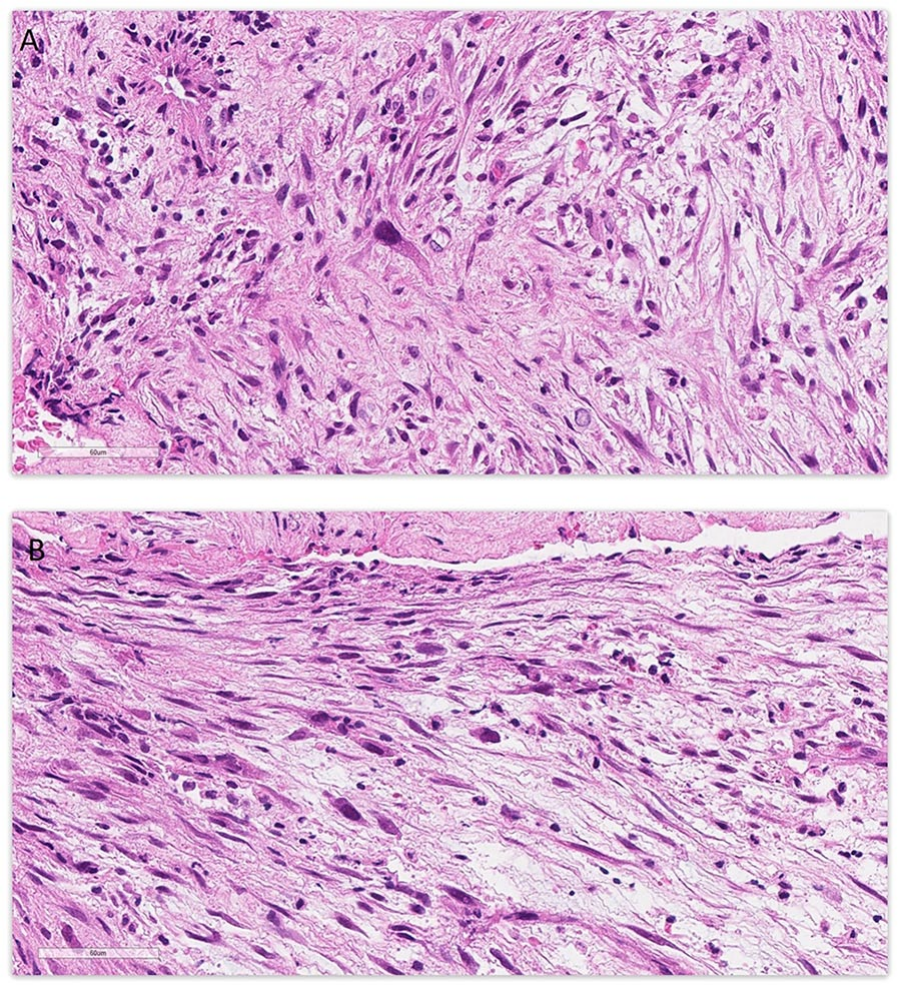
Two areas (A and B) showing focal atypical tumor cells with hyperchromatic large nuclei; Stain: hematoxylin and eosin; Original magnification: 400×; Scale bar: 60 µm.

Pathological examination (Figure 2) revealed a spindle cell neoplasm with low to moderate cellularity. Tumor cells showed a fascicular and irregular storiform growth pattern. Most tumor cells showed bland cytomorphology. In a few areas, more atypical tumor cells were seen (Figure 3), with hyperchromatic nuclei, nuclear pleomorphism, and prominent nucleoli. Extensive coagulative type necrosis (around 75%) was seen. The mitotic count was up to 3 per 10 high power field (HPF). No atypical mitotic figures were seen. The background stroma was fibromyxoid. Prominent thrombus was seen. Tumor cells were mixed with inflammatory infiltrates, mostly neutrophils, eosinophils, and lymphocytes. Plasma cells were not prominent.

Immunohistochemistry (IHC) was performed. Most tumor cells were positive for smooth muscle actin (SMA), but rare atypical tumor cells were negative (Figure 2C). Tumor cells also showed focal positivity for CD31 and desmin. Myogenin, ALK, calretinin, SOX10, and S100 stains were negative. CD34 highlighted endothelial cells only.

**Figure 4.**
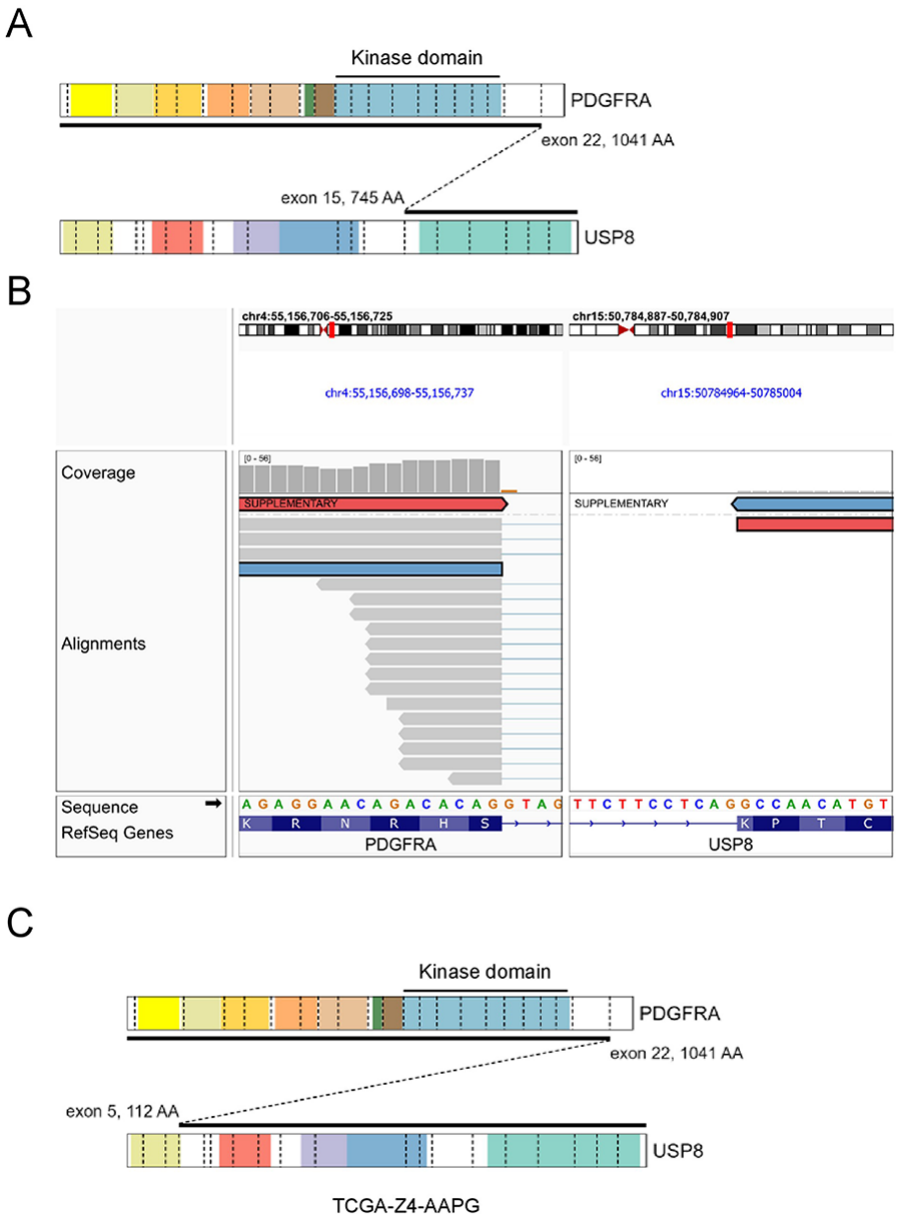
(A) Schematic showing structure of the PDGFRA::USP8 fusion in our case. (B) Genome browser screenshot showing the fusion breakpoints within PDGFRA and USP8. The read pairs highlighted in red and blue support the fusion. (C) Schematic showing structure of the PDGFRA::USP8 fusion from TCGA case TCGAZ4-AAPG. Source: https://pecan.stjude.cloud/proteinpaint.

Illumina TruSight RNA Pan-Cancer next-generation sequencing (NGS) analysis (Figure 4(a) and (b)) was performed. The tumor was found to have an in-frame *PDGFRA*::*USP8* gene fusion. The *PDGFRA* gene breakpoint was at exon 22 (1041 AA). The *USP8* gene breakpoint was at exon 15 (745 AA). The *PDGFRA* kinase domain was preserved. An *ALK* gene mutation was not found. Single nucleotide polymorphism (SNP) array analysis revealed no segmental or whole chromosomal aberrations.

A diagnosis of “unclassified spindle cell neoplasm” was made. Important findings such as *PDGFRA::USP8* gene fusion, necrosis, mitosis, and focal marked atypia were noted, and comment was made that a primary cardiac sarcoma could not be excluded.

Based on the finding of *PDGFRA* gene fusion, option for targeted therapy was discussed with the parents. The parents chose to have the residual tumor resected without additional targeted therapy. Thus, a few months after the first resection, the residual tumor in the ventricular septum was resected (second resection). Patch repair of the resected ventricular septum and patch reconstruction of the septal leaflet of the tricuspid valve were performed. The tumor in the second resection specimen showed similar histopathological features as described above. A likely microscopically positive margin was identified. The case was discussed in multidisciplinary rounds and with the parents, the decision was to closely monitor for tumor recurrence, without additional surgery. The patient was also referred to Cancer Genetics Clinic for testing of Lynch syndrome and Li-Fraumeni syndrome. Sequencing of *EPCAM*, *MLH1*, *MSH2*, *MSH6*, *PMS2*, and *TP53* genes did not identify a clinically significant variant. Eleven months after the second resection, the patient is doing well with no evidence of tumor recurrence.

## Discussion

We report a case of a pediatric primary cardiac spindle cell neoplasm with a *PDGFRA::USP8* gene fusion in the right ventricle, arising from the inlet part of the ventricular septum. This neoplasm showed concerning histological features such as extensive coagulative-type necrosis, cytological atypia, and mitotic activity. IHC showed diffuse staining with smooth muscle actin and focal staining with desmin. RNA sequencing showed a rare *PDGFRA* (exon 22)::*USP8* (exon 15) fusion. The constellation of these findings did not fit into any of the currently known diagnostic categories. Thus a diagnosis of unclassified spindle cell neoplasm was rendered.

Myxofibrosarcoma is probably the closest entity to the tumor in our case. In fact, *PDGFRA::USP8* gene fusion was previously reported from a primary cardiac myxofibrosarcoma in a 5 year old boy. The myxofibrosarcoma was in the left ventricular outflow tract, arising from the right coronary cusp of the aortic valve.^2^ While the tumor in our case did show focal sarcoma-like features, it did not have the lobulated growth pattern or the curvilinear blood vessels typical for myxofibrosarcoma. In addition, the diffuse SMA staining and the lack of segmental chromosomal changes in our case would be unusual for myxofibrosarcoma.

The possibility of inflammatory myofibroblastic tumor (IMT) was considered. The atypical findings in this case were felt more than expected for IMT. IHC stain for ALK was negative, and no *ALK* gene fusion or other known IMT-related genetic changes were detected by TruSight RNA sequencing. Thus IMT was not favored.

Another myofibroblastic tumor, low-grade myofibroblastic sarcoma (LGMFS), was also considered. LGMFS tends to show fibromatosis-like features, which are not present in our case. In addition, our case shows myxoid background, which is not a feature of LGMFS. Thus, LGMFS was not favored.

*PDGFRA::USP8* gene fusion is very rare. To our knowledge, this fusion pair was previously described only twice. One of the 2 cases was the above mentioned pediatric aortic valve myxofibrosarcoma in a 5 year old boy, reported by Krishna et al.^2^ In that case, IHC for PDGFRA protein showed increased activity, and IHC for both SMA and desmin were positive. Details of the breakpoints in the genes were not mentioned in that report. The tumor was successfully resected and Sorafenib was given to reduce the risk of recurrence. The patient had been clinically well for at least 1 year after the surgery.^2^

The other previous case of *PDGFRA::USP8* gene fusion was described by The Cancer Genome Atlas (TCGA) database (TCGA-Z4-AAPG-01A).^3^ The tumor was an undifferentiated pleomorphic sarcoma in retroperitoneum of a 64 year old female. The *PDGFRA* gene breakpoint was in exon 22 (1041 AA). The *USP8* gene breakpoint was in exon 5 (112 AA) (Figure 4(c)). It was an in frame translocation. The patient was diagnosed in 2013. The tumor was completely resected (R0). The patient had been alive and tumor free for at least 16 months following the treatment.

*PDGFRA* is a known oncogene, encoding a cell surface tyrosine kinase receptor protein, and its alterations have been described in many neoplasms. Its mutation can lead to gastrointestinal stromal tumor (GIST), a relatively common mesenchymal neoplasm. It also participates in gene fusions in myeloid or lymphoid neoplasms associated with eosinophilia, with *FIP1L1::PDGFRA* the most common fusion.^4^ *PDGFRA* amplification may also play a role in the oncogenesis of intimal sarcomas and undifferentiated cardiac sarcomas.^5^

*USP8* gene encodes a deubiquitinating enzyme for regulating embryonic stem cell identity and maintaining self-renewal.^6,7^ It has been implicated in oncogenesis in multiple cancers, including lung cancer, cervical cancer, breast cancer, and others. Most of the *USP8*-related cancers are carcinomas.^7^ Its role in oncogenesis of mesenchymal neoplasms is uncertain.

There are numerous molecular diagnostic assays available to the pathologist, and selecting the most appropriate molecular test can sometimes be a challenge. In our center, molecular diagnostics for sarcoma usually starts with our NanoString assay (covers 34 common fusion transcripts for pediatric soft tissue tumors). If non-diagnostic, or if the tumor appears sufficiently uncommon or rare, we use our Illumina TruSight RNA Pan-Cancer Panel, a targeted RNA-based NGS assay covering 1385 oncology genes. This NGS-based assay detects both gene fusions as well as single-nucleotide-variants (SNVs). In this case, since the tumor appeared to be a rare tumor, we used the Illumina TruSight assay up front and discovered the *PDGFRA::USP8* gene fusion.

In summary, we report a case of pediatric primary cardiac unclassified spindle cell neoplasm with a very rare *PDGFRA::USP8* gene fusion. To our knowledge, this is the first reported case of spindle cell neoplasm with *PDGFRA::USP8* gene fusion arising from right cardiac ventricle. Pediatric cardiac sarcomas are very rare. Molecular analysis of pediatric cardiac sarcomas is strongly recommended for better classification and therapeutic decisions for these tumors.
